# Male–female disparity in clinical features and significance of mild vertebral fractures in community-dwelling residents aged 50 and over

**DOI:** 10.1038/s41598-024-56379-6

**Published:** 2024-03-07

**Authors:** Shota Ikegami, Masashi Uehara, Ryosuke Tokida, Hikaru Nishimura, Noriko Sakai, Hiroshi Horiuchi, Hiroyuki Kato, Jun Takahashi

**Affiliations:** 1 Department of Orthopaedic Surgery, Shinshu University School of Medicine, 3-1-1 Asahi, Matsumoto, Nagano 390-8621 Japan; 2 Rehabilitation Center, Shinshu University Hospital, 3-1-1 Asahi, Matsumoto, Nagano 390-8621 Japan; 3 Department of Orthopaedic Surgery, New Life Hospital, 851 Obuse, Kamitakai-gun, Nagano, 381-0295 Japan

**Keywords:** Epidemiology, Population screening, Physical examination

## Abstract

This investigation examined the clinical implications of mild vertebral fractures in older community-dwelling residents. Focusing on the locomotion health of older individuals, the earlier reported Obuse study enrolled 415 randomly sampled Japanese residents aged between 50 and 89 years, 411 of whom underwent X-ray evaluations for pre-existing vertebral fractures. A blinded assessment of vertebral fractures based on Genant’s criteria was conducted on the T5-L5 spine for rating on a severity scale. Grade 1 mild fractures were not linked to age in males, but increased with aging in females. Female participants had fewer Grade 1 and 2 fractures (P = 0.003 and 0.035, respectively) but more Grade 3 fractures (P = 0.013) than did males independently of age (Grade 1, 2, and 3: 25%, 16%, and 9% in females and 40%, 22%, and 6% in males, respectively). Weak negative correlations were observed between the number of fractures and bone mineral density in females for all fracture grades (Spearman’s rho: 0.23 to 0.36, P < 0.05). Our study showed that Grade 1 mild vertebral fractures in males lacked pathological significance, while in females they potentially indicated fragility fractures and were related to poor lumbopelvic alignment.

## Introduction

Fragility fractures due to osteoporosis that are caused by low-energy trauma occur frequently in older adults, leading to declines in quality of life (QOL) as well as activities of daily living (ADL) and requiring nursing care^1,2^. Accordingly, moderate to severe vertebral fractures are targets for non-operative and operative therapies. The primary aims of treatment are pain relief, restoration of vertebral height and alignment, and bone healing, with the ultimate goal of assisting QOL and ADL recovery^3^. The need for treating osteoporosis, which is an indirect cause of such fractures, has also been emphasized^4^.

In clinical practice, physicians sometimes encounter radiographs of mild vertebral body wedging deformation without any particular complaints. Especially with no suspicion of bone fragility and if the patient is young, vertebral body wedging is unlikely to equate to the osteoporotic vertebral body fractures seen in older people. Morphologically, however, the deformities are difficult to clearly distinguish from old traumatic fracture deformity, and so have been termed “mild vertebral fracture” in this paper for convenience. Although the frequency and clinical significance of mild vertebral fractures have been investigated in Europe^5,6^, few studies exist in Japanese community-dwelling older people^7^. We therefore sought to address this issue by means of the Obuse Study, a resident survey project. The present investigation aimed to determine the sex disparities and clinical significance of mild vertebral fractures in older randomly selected town residents.

## Materials and methods

### Creation of a randomly sampled resident cohort for epidemiological survey

The Obuse study is a comprehensive investigation of the locomotion health of community-dwelling older people. Obuse is the name of the cooperating suburban town located in a central inland area of Japan, with a population of approximately 10,000 people. In the establishment of this new population study of the Japanese, we employed random sampling from the basic town resident registry to minimize selection bias and obtain a cohort representative of the general population. Residents between the age of 50 and 89 years were randomly sampled to construct a 415-participant cohort named the Obuse study cohort. Among them, 411 individuals who were able to stand unassisted and did not have any spinal implants were subjected to analysis. This study was approved by the investigational review board of Shinshu University Life Science and Medical Sciences Research Ethics Committee (approval number: 2792). All methods were performed in accordance with the relevant guidelines and regulations in addition to STROBE guidelines. All participants provided written informed consent for study participation.

### X-ray evaluation of pre-existing vertebral fractures and spinal alignment

**Figure 1:**
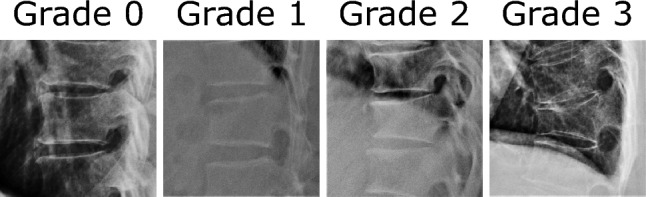
Grading of vertebral fractures by a semi-quantitative method^8^.

The tag of “fracture” in this paper was based solely on imaging assessments and not on the clinical definition. All study participants underwent lateral X-ray photography. Two board-certified spine surgeons assessed for the presence and Grade of pre-existing morphological vertebral fractures in the T5 to L4 spine in a blinded manner according to a semi-quantitative method^8^. This grading has been commonly used in the clinical setting^9,10^, with Grade 0 classified as a non-fracture, Grade 1 as a mild fracture with vertebral body height reduced by 20–25%, Grade 2 as a moderate fracture with 25–40% of vertebral body height reduction, and Grade 3 as a severe fracture with 40% or more vertebral body height reduction (Fig. 1). Vertebrae were judged as fractured in cases where both evaluators determined a fracture. The final grading was considered the less severe Grade between the two examiners. The inter-rater reliability of each set of four was 0.925 for T5–T8, 0.934 for T9–T12, and 0.960 for L1–L4.

Sagittal spinal alignment parameters, including sagittal vertical axis (SVA) and global tilt (GT) as indicators of subcervical total spinal alignment, thoracic kyphosis (TK: kyphotic angle between the T5 superior endplate and the T12 inferior endplate), lumbar lordosis (LL: lordotic angle between the L1 superior endplate and the sacral plate), and pelvic tilt (PT: angle subtended by a vertical reference line originating from the center of the femoral head and the midpoint of the sacral plate) as indicators of local alignment, were also measured in radiographs. The average values of measurements by the two evaluators and a trained staff member were used for each parameter. The inter-rater reliability for each parameter was 0.95 for SVA, 0.71 for GT, 0.92 for TK, and 0.89 for PT. The inter-rater reliability for LL was moderate at 0.65. In our previous report^11^, the inter-rater reliability for sacral slope was 0.48 and comparatively lower than those for other spinal parameters, as was the case for LL in the present study. Thus, variable interpretation of S1 endplate shape, which is frequently degenerated in the Japanese elderly population, may have lowered reliability among the evaluators.

### Relevant information regarding vertebral fractures

The participants were measured for bone mineral density (BMD) at the L2–L4 spine, bilateral total hip, and femoral neck using Dual-energy X-ray absorptiometry (Prodigy; GE Healthcare, Chicago, IL). Femoral (femoral neck and total hip) BMD was assessed as the mean value of both legs. The experience of past vertebral fracture was checked by interview.

### Statistical analyses

The prevalence of morphometric vertebral fractures was tabulated according to sex (male or female) and age group (50’s to 80’s). The association between the number of vertebral fractures by fracture Grade and age or BMD was evaluated by Spearman’s correlation coefficient. The relationship between the number of fractures and sex was assessed by univariate and multivariate Poisson regression analysis, with the number of vertebral fractures by Grade as the response variable, sex as an explanatory variable, and age group as a covariate in multivariate analysis. Sensitivity and specificity were calculated for the coincidence of the recognized experience of vertebral fracture by interview and the presence of morphological vertebral fractures by X-ray assessment. The relationship between sagittal spinal alignment and the number of vertebral fractures was evaluated by multiple linear regression analysis, with the alignment parameter value as the response variable, the number of vertebral fractures included in the range of alignment measurement spinal level by fracture Grade (Grade 1 or 2/3) as an explanatory variable, and age group as a covariate. The relationship between QOL scores, i.e., mental component summary (MCS) and physical component summary (PCS) scores of the SF-8, and the number of vertebral fractures was analyzed in the same way as for sagittal spinal alignment. BMD, sagittal spinal alignment parameters, and QOL scores of the participants in this study are summarized in Supplementary Tables 1, 2 and 3, respectively. Statistical analyses were carried out using the statistical package R, version 3.6.3 (available at http://www.r-project.org). The level of significance was set at P < 0.05.

## Results

Table 1 summarizes the baseline characteristics of the Obuse study cohort. The 411 participants were almost uniformly divided into each sex/age decade category. While average height and weight tended to decrease with age, body mass index did not. Osteoporosis medications tended to be used less frequently in males. Older females were more likely to be taking some type of osteoporosis drug, with 17% and 34% of participants in their 70 s and 80 s, respectively, receiving osteoporosis treatment.

**Table 1:** Baseline characteristics of the Obuse study cohort.

Sex	Age (years)	Number	Height (cm)	Weight (kg)	BMI (kg/m^2^)	Osteoporosis medication (number and %)
Male	50’s	50	171.8 (6.0)	67.1 (9.1)	22.7 (2.9)	0 (0.0%)
	60’s	53	166.7 (4.7)	66.9 (7.7)	24.1 (2.7)	1 (1.9%)
	70’s	55	163.2 (5.0)	60.0 (10.3)	22.5 (3.4)	2 (3.6%)
	80’s	45	160.1 (5.7)	57.5 (8.5)	22.4 (2.8)	2 (4.4%)
	All	203	165.6 (6.8)	63.0 (9.8)	22.9 (3.0)	5 (2.5%)
Female	50’s	47	158.1 (4.9)	55.4 (9.0)	22.2 (3.8)	1 (2.1%)
	60’s	61	152.8 (5.4)	52.2 (7.6)	22.3 (2.8)	2 (3.3%)
	70’s	53	149.7 (5.3)	50.5 (7.9)	22.5 (3.2)	9 (17.0%)
	80’s	47	144.5 (5.9)	48.1 (7.8)	23.0 (3.3)	16 (34.0%)
	All	208	151.4 (7.1)	51.6 (8.4)	22.5 (3.2)	28 (13.5%)

Values represent the mean (standard deviation).

*BMI* body mass index.

### Association of vertebral fractures with age and sex

**Figure 2:**
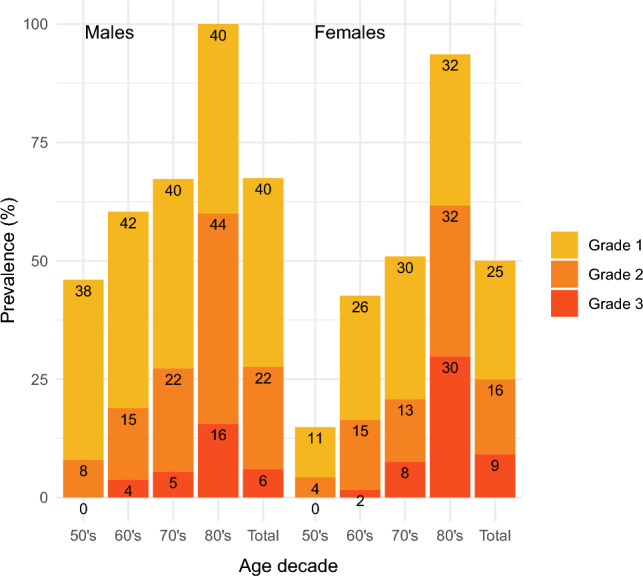
Prevalence of morphometric vertebral fractures.

The prevalence of vertebral fractures by Grade is presented in Fig. 2. The correlation coefficient between age and the number of fractures for each Grade is shown in Table 2. Approximately 40% of male participants had a Grade 1 fracture among all age groups, with no significant correlation between the number of Grade 1 fractures and age. Grade 1 fractures in females showed an overall prevalence of 25%. We observed a significant weak positive correlation between age and the number of Grade 1 fractures. Grade 2/3 fractures tended to be more prevalent in older males, with a significant weak positive correlation between the number of fractures and age.

**Table 2:** Correlation between the number of vertebral fractures by Grade and age.

	All	Grade 1	Grade 2	Grade 3	Grade 2/3
Male	0.20*	− 0.02	0.30*	0.22*	0.35*
Female	0.37*	0.23*	0.26*	0.36*	0.36*

Values are expressed as Spearman correlation coefficients.

*P < 0.05.

The number of Grade 1 and 2 fractures was significantly lower in females than in males, while the number of Grade 3 fractures was significantly higher (Table 3). These relationships were independent of age.

**Table 3:** Association between the number of fractures and sex.

Fracture	Crude		Adjusted with age	
Grade	Rate ratio (95% CI)	P value	Rate ratio (95% CI)	P value
All	0.77 (0.62–0.96)	0.018*	0.76 (0.61–0.95)	0.014*
Grade 1	0.64 (0.47–0.86)	0.004*	0.64 (0.47–0.86)	0.003*
Grade 2	0.67 (0.45–0.98)	0.042*	0.66 (0.45–0.97)	0.035*
Grade 3	2.33 (1.25–4.61)	0.011*	2.28 (1.22–4.51)	0.013*

Values represent the rate ratio of female to male for the number of vertebral fractures each person has.

*CI* confidence interval.

*P < 0.05.

### Association of vertebral fractures with bone mineral density

We detected significant weak negative correlations for the number of vertebral fractures with femoral BMD in both sexes (Table 4). Similar, but weaker, associations were also seen for lumbar BMD. More detailed inspection revealed that higher Grade fractures were associated with BMD, but lower Grade fractures, especially in males, were not. There was no correlation between the number of Grade 1 fractures and BMD in males. However, we observed a significant, but very weak, negative correlation between the number of Grade 1 fractures and femoral BMD in females. The number of Grade 2 and Grade 3 fractures showed significant but weak negative correlations with femoral BMD in both sexes.

**Table 4:** Correlation between the number of vertebral fractures by Grade and bone mineral density.

Sex	Site	All	Grade 1	Grade 2	Grade 3
Male	L2–L4	− 0.15*	− 0.06	− 0.07	− 0.22*
	Femoral neck	− 0.24*	− 0.10	− 0.16*	− 0.26*
	Total hip	− 0.23*	− 0.06	− 0.19*	− 0.26*
Female	L2–L4	− 0.18*	− 0.04	− 0.10	− 0.21*
	Femoral neck	− 0.30*	− 0.16*	− 0.16*	− 0.25*
	Total hip	− 0.30*	− 0.18*	− 0.18*	− 0.26*

*P < 0.05.

### Awareness of having experienced vertebral fractures

Table 5 shows the relationship of the recognized experience of vertebral fracture by interview and the presence of morphometric vertebral fractures by X-ray assessment. Minor vertebral fractures were generally not recognized as injury experiences. The sensitivity of subjects with Grade 3 fractures who were aware of their injury experience was approximately 30%, and was even lower for combined Grade 2 and 3 fractures. High specificity (more than 98%) was noted for those who had no fracture and reported no injury experience.

**Table 5:** Coincidence of the recognized experience of vertebral fracture by interview and the presence of morphometric vertebral fractures by X-ray assessment.

Fracture grade	Sensitivity (%)	Specificity (%)
Grade 1, 2, 3	8.1	100.0
Grade 2, 3	14.9	99.7
Grade 3	32.3	98.7

Values are the sensitivity and specificity of interviews about past vertebral fractures to detect pre-existing morphological fractures.

### Impact of vertebral fractures on spinal sagittal alignment

**Figure 3:**
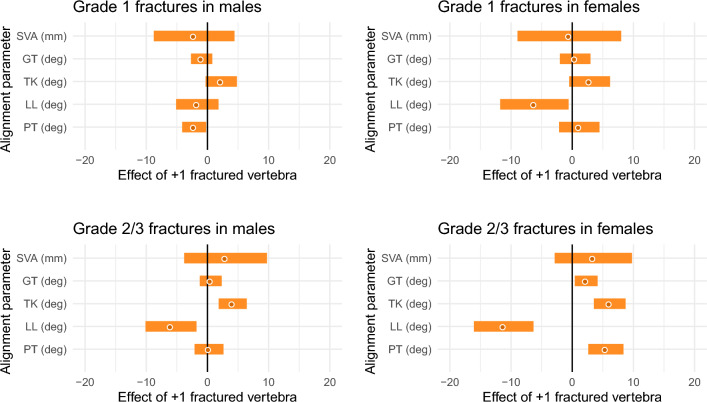
Relationship between sagittal spinal alignment and the number of vertebral fractures. The effect values were adjusted for age group. Bands indicate the 95% confidence intervals. *SVA* sagittal vertical axis, *GT* global tilt, *TK* thoracic kyphosis, *LL* lumbar lordosis, *PT* pelvic tilt, *deg* degrees.

The respective number of vertebral fractures had more obvious effects on sagittal spinal alignment for Grade 2/3 fractures than for Grade 1 fractures and in females than in males (Fig. 3). In male subjects, Grade 1 vertebral fractures had no apparent effect on sagittal spinal alignment, although PT was 2.2 degrees (95% confidence interval [95% CI] 0.2 to 4.1, P = 0.02) smaller for each additional fracture. Grade 2/3 vertebral fractures had a different trend than did Grade 1 fractures and were significantly associated with larger TK (+ 4.1 degrees per 1 vertebra, 95% CI 1.9 to 6.4, P < 0.01) and smaller LL (− 5.9 degrees per 1 vertebra, 95% CI − 10.1 to − 1.8, P < 0.01). In females, Grade 1 vertebral fractures were significantly associated with smaller LL (− 6.2 degrees per 1 vertebra, 95% CI − 11.7 to − 0.6, P = 0.03), while Grade 2/3 vertebral fractures were associated with larger GT (+ 2.3 degrees per 1 vertebra, 95% CI 0.4 to 4.1, P = 0.01), TK (+ 6.1 degrees per 1 vertebra, 95% CI 3.5 to 8.7, P < 0.01), and PT (+ 5.5 degrees per 1 vertebra, 95% CI 2.6 to 8.4, P < 0.01), as well as smaller LL (− 11.2 per 1 vertebra, 95% CI − 16.1 to − 6.4, P < 0.01).

### Relationship between the number of fractured vertebrae and QOL

**Figure 4:**
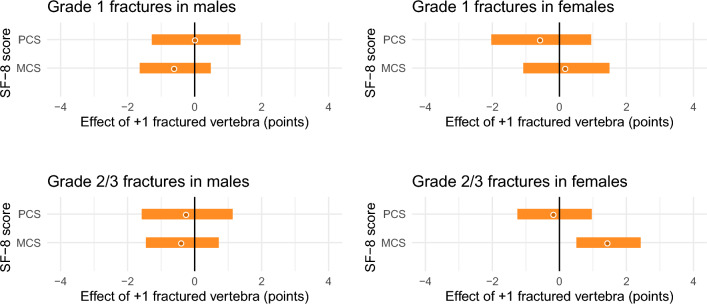
Relationship between quality of life and the number of vertebral fractures. The effect values were adjusted for age group. Bands indicate the 95% confidence intervals. *PCS* physical component summary score, *MCS* mental component summary score.

Figure 4 shows the impact of the number of fractured vertebrae on the PCS and MCS scores of the SF-8 questionnaire. We saw no clear association between Grade 1 fractures and QOL scores in either sex. No significant association was found between the number of Grade 2/3 fractures and QOL scores in males, but in females, MCS score unexpectedly increased with the number of Grade 2/3 fractures (+ 1.5 points per 1 vertebra, 95% CI 0.5 to 2.4, P < 0.01).

## Discussion

In this study, Grade 1 mild vertebral fractures in males were present irrespectively of age or BMD. On the other hand, such fractures increased with age in females and were correlated with hip BMD. High Grade fractures affected regional alignment, the effect of which being more pronounced in the lumbopelvic region of female subjects. In females, pre-existing vertebral body fractures had the characteristics of fragility fractures even with mild wedging deformation that increased with Grade. In males, however, mild wedging deformation did not exhibit the characteristics of fragility fractures. The real-world implications of our results are that while mild vertebral fractures in males need not be treated as clinical fractures, such findings cannot be ignored in females to prevent future fragility fractures.

We observed that the prevalence of morphological vertebral fractures did not differ significantly between sexes, and that the self-perception rate of morphological fractures was generally very low. A previous report found the prevalence of radiographic vertebral fractures in subjects older than 65 years to range from 15 to 25% in females and 7.3% to 18% in males in the Eastern Mediterranean region^12^. In the Middle East, prevalent vertebral fractures existed in 13.3–20.2% of females (mean age: 58–74 years) and 10–14% of males (mean age: 62–74 years), excluding Grade 1 fractures^13^. Our results revealed much higher prevalence rates (Fig. 2). The reasons for this difference include regional and racial differences as well as possible discrepancies in the thresholds for evaluation of vertebral fractures. In general, vertebral body fractures are considered to be of important clinical significance as fragility fractures in older people. Clinicians understand that vertebral fractures occur in conjunction with decreased BMD^14^, and that wedged fractures worsen spinal alignment^15^ and reduce QOL^16^. However, the results of the current study did not support those consensuses. This discrepancy may be explained by our survey targeting the general healthy population rather than osteoporosis patients; implicit morphological vertebral fractures far outnumber vertebral fractures that require recognition and treatment.

Lumbar spine BMD and hip BMD are both relevant factors associated with vertebral body fractures^12,17^. While lumbar spine BMD is sometimes overestimated due to spondylotic change, hip BMD is regarded as a relatively accurate assessment of bone strength due to differences in the frequency of degeneration^18^. In the current study, Grade 1 mild vertebral fractures in males did not correlate with hip BMD, and the prevalence of factures was approximately 40% regardless of age in males over 50 years of age. These results suggest that treatments to increase BMD may not prevent Grade 1 fractures in males. Strict morphological evaluation has revealed that more males than females did not have osteoporosis even with vertebral body deformity^6^. However, in females, Grade 1 fractures were also correlated with BMD, and their prevalence increased with age. Grade 1 fractures may therefore be preventable with osteoporosis treatments in females.

Our findings suggest that whereas mild vertebral fractures in males may not demonstrably influence posture, their impact on spinal alignment in females cannot be ignored. In the general population over 50 years old, LL decreases and PT increases with age^11,19,20^, and this lumbar-pelvic alignment feature is more pronounced in females. In our cohort, Grade 2/3 morphological lumbar vertebral body fractures in female subjects were associated with lumbopelvic alignment even after excluding the effects of age. Wedge-shaped vertebral morphological changes might have had a direct effect on LL and a compensatory effect by PT. In addition, Grade 1 fractures affected LL in female subjects. Given that older females are more likely to have Grade 1, 2, and 3 fractures, the results of this study may help explain the mechanism of alignment features in females mentioned in previous reports^15–17^. On the other hand, Grade 2 and 3 fractures contributed to LL reduction in males, with Grade 1 fractures exerting no clear effect. The deviation of lumbo-pelvic spinal alignment in males over 50 years of age among community-dwelling residents was found to be smaller than that in females of the same age group^11^, which might have contributed to the unclear impact of vertebral fractures on spinal alignment in male participants. If alignment change according to aging is preventable by osteoporosis therapies, the health condition of community-dwelling older people might be preserved, especially for females, since thoraco-pelvic spinal alignment deterioration has been associated with worsened cervical spinal alignment^21^, lower physical performance^22^, and mild cognitive decline^23^.

Lastly, vertebral body fractures were not found to be convincingly associated with QOL as indicated by SF-8 scores. Previous reports have linked clinical vertebral fractures with lower QOL^12^. Our results uncovered no clear association for physical QOL, although psychologically, bone fractures were associated with higher QOL in females. The reasons for this inconsistent result may be that we focused on morphological fractures rather than clinical fractures in addition to underlying differences in the study population. Our subjects were community-dwelling and essentially healthy residents. In such a population, the impact of fractures on QOL may not be present as in patient settings with more severe influencing factors.

Several constraints may have limited this study. First, the possibility of bias between the observers could have impacted the results. Furthermore, the cross-sectional design of the study prevented the determination of causal links between vertebral fractures and other observed factors; longitudinal investigations are required to confirm any conclusions on the changes reliant on aging. Moreover, we did not make preliminary computations about the necessary sample size to ensure sufficient clinical variation for broad conclusions. As a result, our findings may have been shaped by the type and frequency of vertebral fractures and spinal deformities in the people who chose to participate. Regional features may have also restricted this study as our sampling was limited to a fairly small town. Even though this facilitated the present epidemiological survey and reduced resident displacement, our findings might not be representative of residents in urban areas.

## Conclusion

Grade 1 mild vertebral fractures in males are likely not fragility fractures and have no pathological significance. On the other hand, such fractures in females are possible fragility fractures and associated with poor alignment in the lumbar spine. Grade 2 and 3 lumbar fractures have a clear impact on lumbopelvic alignment, especially in females, and may contribute to sex differences in lumbopelvic alignment changes in older people. Clinically, whereas Grade 1 mild vertebral fractures may not require treatment in males, intervention is advisable in females.

### Supplementary Information

Supplementary Table 1.

Supplementary Table 2.

Supplementary Table 3.

## Data Availability

The datasets generated and/or analyzed during the current study are available in the Zenodo repository (10.5281/zenodo.10276777).
