# Supplementary material for: Male–female disparity in clinical features and significance of mild vertebral fractures in community-dwelling residents aged 50 and over
Source: Sci Rep. 2024 Mar 7;14:5602. doi: 10.1038/s41598-024-56379-6 (PMC10920731; doi:10.1038/s41598-024-56379-6)
Supplement: Supplementary file 1 — Supplementary Table 1. [file 41598_2024_56379_MOESM1_ESM.docx]

Supplemental table 1. Summary of bone mineral density.

| Sex | Age (years) | L2-L4 (g/cm^2^) | Femoral neck (g/cm^2^) | Total hip (g/cm^2^) |
| --- | --- | --- | --- | --- |
| Male | 50's | 1.173 (0.183) | 0.900 (0.134) | 0.959 (0.129) |
|  | 60's | 1.282 (0.221) | 0.905 (0.114) | 0.987 (0.129) |
|  | 70's | 1.347 (0.273) | 0.884 (0.121) | 0.969 (0.148) |
|  | 80's | 1.273 (0.286) | 0.803 (0.145) | 0.866 (0.149) |
|  | All | 1.271 (0.250) | 0.875 (0.133) | 0.948 (0.145) |
|  |  |  |  |  |
| Female | 50's | 1.092 (0.175) | 0.798 (0.108) | 0.870 (0.128) |
|  | 60's | 1.024 (0.182) | 0.761 (0.102) | 0.836 (0.106) |
|  | 70's | 1.023 (0.207) | 0.719 (0.117) | 0.807 (0.120) |
|  | 80's | 0.996 (0.201) | 0.672 (0.095) | 0.711 (0.099) |
|  | All | 1.033 (0.193) | 0.739 (0.115) | 0.808 (0.126) |

*Note:* Values represent the mean (standard deviation).
