# Supplementary material for: Male–female disparity in clinical features and significance of mild vertebral fractures in community-dwelling residents aged 50 and over
Source: Sci Rep. 2024 Mar 7;14:5602. doi: 10.1038/s41598-024-56379-6 (PMC10920731; doi:10.1038/s41598-024-56379-6)
Supplement: Supplementary file 2 — Supplementary Table 2. [file 41598_2024_56379_MOESM2_ESM.docx]

Supplemental table 2. Summary of sagittal spinal alignment parameters.

| Sex | Age (years) | SVA (mm) | GT (degrees) | TK (degrees) | LL (degrees) | PT (degrees) |
| --- | --- | --- | --- | --- | --- | --- |
| Male | 50's | 6 (26) | 13 (7) | 25 (8) | 44 (11) | 12 (6) |
|  | 60's | 9 (38) | 17 (11) | 29 (8) | 45 (13) | 14 (8) |
|  | 70's | 22 (30) | 19 (8) | 31 (10) | 45 (13) | 16 (6) |
|  | 80's | 57 (49) | 29 (12) | 31 (13) | 38 (12) | 21 (7) |
|  | All | 22 (41) | 19 (11) | 29 (10) | 43 (12) | 16 (7) |
|  |  |  |  |  |  |  |
| Female | 50's | -5 (26) | 14 (8) | 27 (9) | 51 (11) | 14 (6) |
|  | 60's | 5 (30) | 19 (11) | 31 (10) | 47 (14) | 17 (8) |
|  | 70's | 31 (36) | 26 (11) | 30 (11) | 42 (14) | 23 (10) |
|  | 80's | 61 (60) | 36 (16) | 33 (19) | 38 (21) | 27 (11) |
|  | All | 22 (47) | 23 (14) | 30 (12) | 45 (16) | 20 (10) |

*Note:* Values represent the mean (standard deviation).

*Abbreviations:* SVA, sagittal vertical axis; GT, global tilt; TK, thoracic kyphosis; LL, lumbar lordosis; PT, pelvic tilt.
