# Supplementary material for: Male–female disparity in clinical features and significance of mild vertebral fractures in community-dwelling residents aged 50 and over
Source: Sci Rep. 2024 Mar 7;14:5602. doi: 10.1038/s41598-024-56379-6 (PMC10920731; doi:10.1038/s41598-024-56379-6)
Supplement: Supplementary file 3 — Supplementary Table 3. [file 41598_2024_56379_MOESM3_ESM.docx]

Supplemental table 3. Summary of SF-8 scores.

| Sex | Age (years) | PCS (points) | MCS (points) |
| --- | --- | --- | --- |
| Male | 50's | 50 (6) | 49 (6) |
|  | 60's | 50 (7) | 50 (5) |
|  | 70's | 47 (7) | 50 (5) |
|  | 80's | 44 (9) | 52 (7) |
|  | All | 48 (8) | 50 (6) |
|  |  |  |  |
| Female | 50's | 51 (6) | 47 (7) |
|  | 60's | 50 (6) | 50 (5) |
|  | 70's | 46 (7) | 50 (6) |
|  | 80's | 42 (9) | 50 (7) |
|  | All | 48 (8) | 49 (6) |

*Note:* Values represent the mean (standard deviation).

*Abbreviations:* PCS, physical component summary score; MCS, mental component summary score.
